# Supplementary material for: Membrane Tension Regulation is Required for Wound Repair
Source: Adv Sci (Weinh). 2024 Oct 3;11(48):2402317. doi: 10.1002/advs.202402317 (PMC11672324; doi:10.1002/advs.202402317)
Supplement: Supplementary file 1 — Supporting Information [file ADVS-11-2402317-s011.pdf]

ADVANCED SCIENCE
Open Access

Supporting Information

for *Adv. Sci.*, DOI 10.1002/advs.202402317

Membrane Tension Regulation is Required for Wound Repair

Nikita Raj, Martin S. Weiß, Bart E. Vos, Sarah Weischer, Frauke Brinkmann, Timo Betz, Britta Trappmann and Volker Gerke**

Supporting Information

Membrane Tension Regulation is Required for Wound Repair

*Nikita Raj**, *Martin S. Weiß, Bart E. Vos, Sarah Weischer, Frauke Brinkmann, Timo Betz, Britta Trappmann, Volker Gerke**

This PDF file includes:

Figs. S1 to S11
Captions for Videos S1 to S9

Other Supporting Information for this manuscript include the following:

Videos S1 to S9

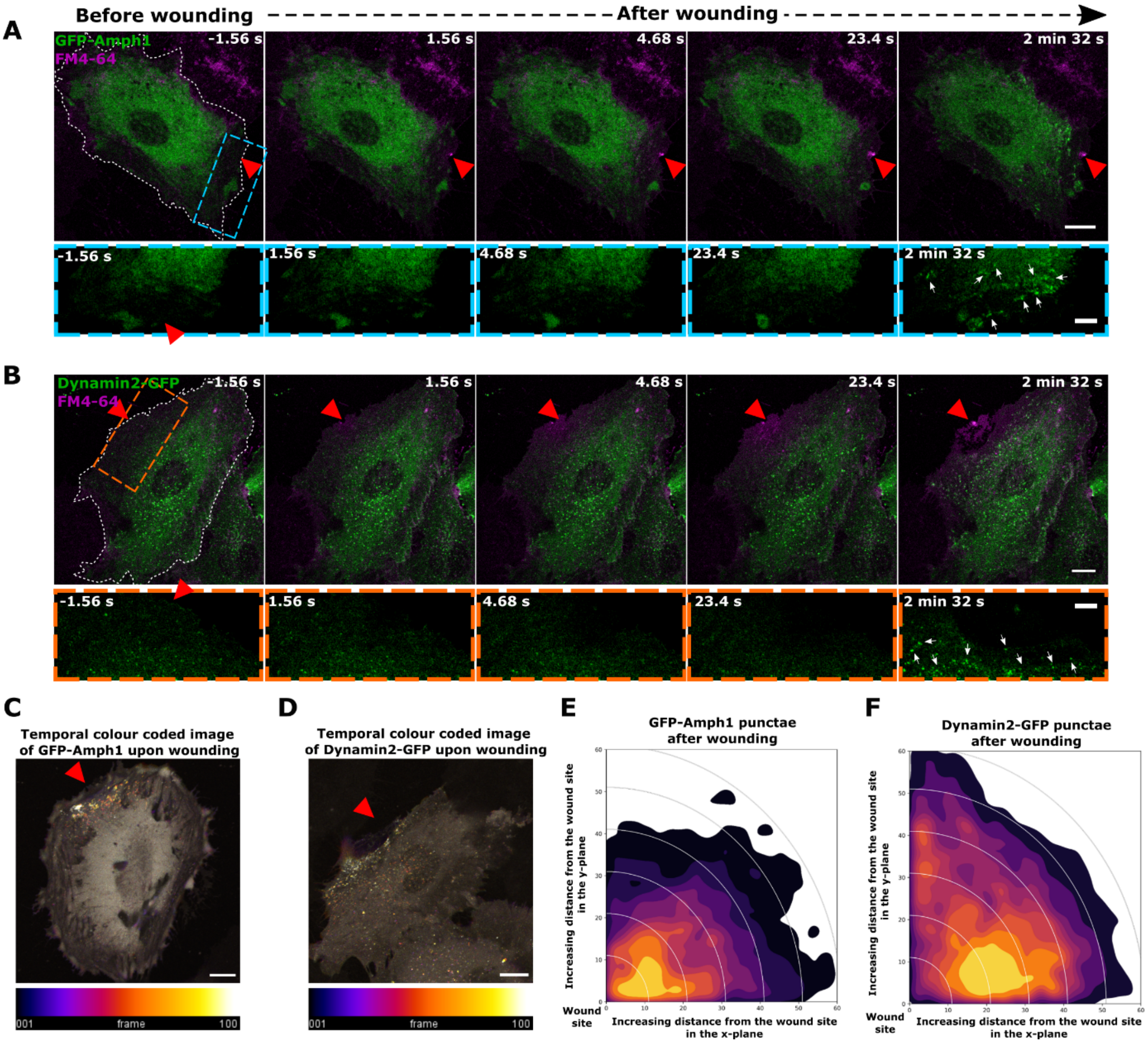

Figure S1. Enhanced clathrin-mediated endocytosis does not occur during wound resealing in HUVEC, but only at later stages of wound repair. (**A - B**) HUVEC were transfected with markers of clathrin-mediated endocytosis (CME), EGFP-Amphiphysin-1 (labelled as GFP-Amph1) (**A**) or Dynamin-2-EGFP (labelled as Dynamin2-GFP) (**B**) (both displayed in green) and subjected to laser injury in the presence of the membrane-impermeable wounding dye, FM4-64 (magenta). Representative images of the same cell before and after wounding are shown here to detect the resealing response and corresponding dynamics of the endocytic proteins. Boxed areas below show higher magnifications around the wound site at each time point for EGFP-Amphiphysin-1 (blue box) and Dynamin-2-EGFP (orange box). Note that successful resealing was observed around 20-30 *s* after wounding, as detected by the containment of the FM4-64 dye influx around the site of wounding (time point 4 for both panels). No changes in GFP-Amph1 (A) or Dynamin-2-EGFP (B) were observed during this time, but a significant enhancement of both markers as punctate structures occurred at a later time point after resealing (last time point for both panels). Red triangles in the initial time point

indicate wound sites, and in all other panels point to the FM4-64 signal containment around the wound site. White dashes, wounded cells. White arrows indicate the endocytic punctae formed around the wound site, which are absent in the previous frames. Scale bars, 10 μm and for zooms, 5 μm. (**C and D**) Temporally colour-coded images of CME events upon wounding of HUVEC expressing GFP-Amph1 (**C**) or Dynamin2-GFP (**D**) (corresponding to images in Figure 1A and B, respectively). The images are generated from all time frames and show the endocytic punctae formation with respect to time after wounding, represented in the colour scale below each image (with frame 1 = -1.56 s and frame 100 corresponding to 2 min 33 s after wounding). Apart from the restricted localization of CME punctae around the wound site, note that the punctae close to the wound are brighter coloured indicating that they are formed in the later stages of repair. Red triangles, wound sites, and scale bars, 10 μm. (**E and F**) Density plot representations of GFP-Amph1 punctae (**E**) or Dynamin2-GFP punctae (**F**) formed in HUVEC after wounding. Here, the distribution of the punctae from all time frames is plotted based on the distance to the wound site, with the wound co-ordinates being set to (0, 0) (origin of the graph). Note that the majority of the punctae formed by both endocytic proteins after laser injury are close to the wound site, suggesting a wounding-induced upregulated endocytic response, that occurs distinct from steady-state endocytosis events that happen all over the cell across time. Punctae counts in the frames before wounding are also included here, which possibly explains the widespread distribution of Dynamin-2 structures owing to its punctate pre-wounding localization all over the cell unlike more cytosolically localized GFP-Amph1. Mean endocytic punctae counts are plotted here from 18 – 25 cells compiled from 3 biological experiments.

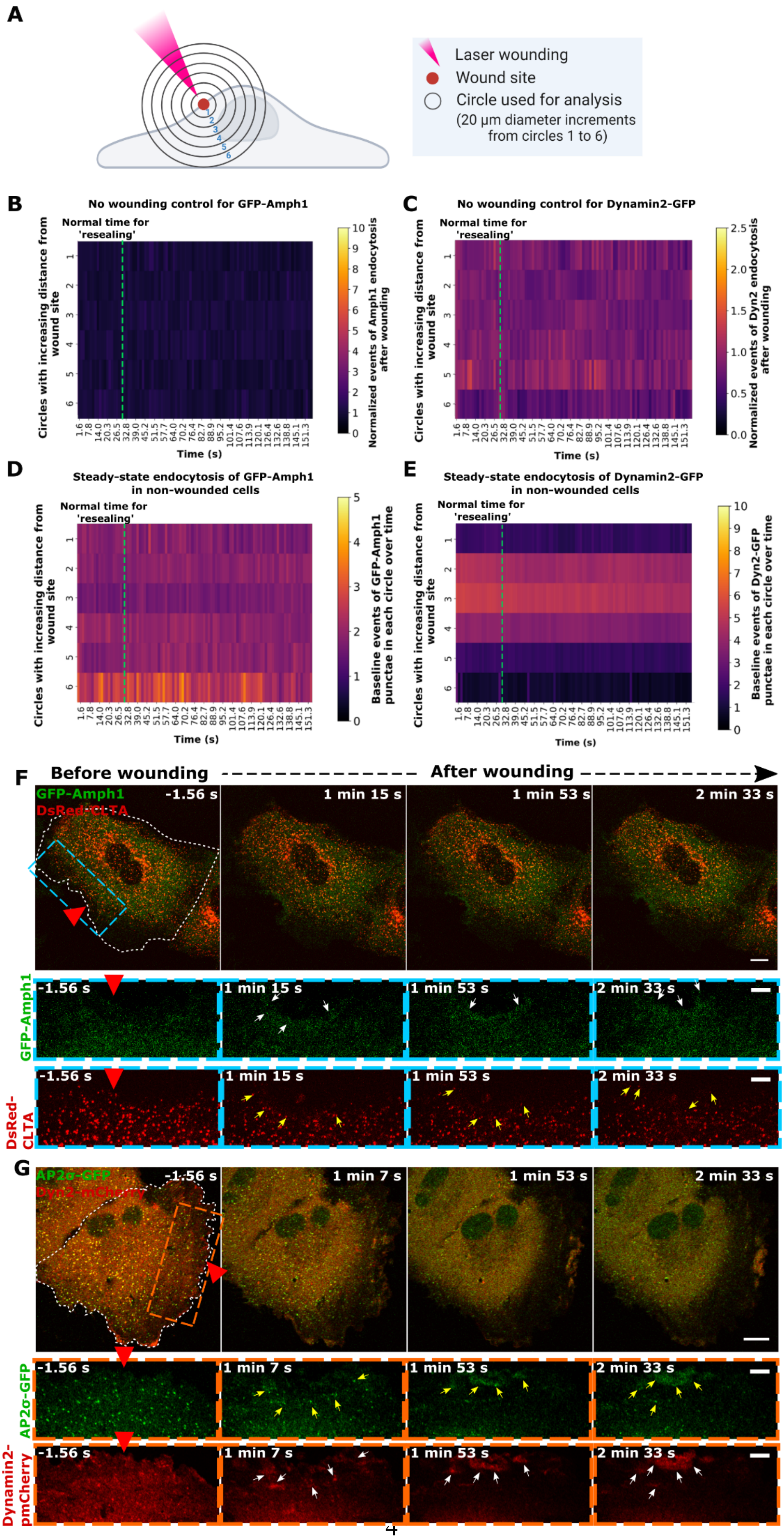
A
Laser wounding
Wound site
Circle used for analysis
(20 µm diameter increments from circles 1 to 6)
B
No wounding control for GFP-Amph1
Normal time for 'resealing'
Circles with increasing distance from wound site
Normalized events of Amph1 endocytosis after wounding
Time (s)
C
No wounding control for Dynamin2-GFP
Normal time for 'resealing'
Circles with increasing distance from wound site
Normalized events of Dyn2 endocytosis after wounding
Time (s)
D
Steady-state endocytosis of GFP-Amph1 in non-wounded cells
Normal time for 'resealing'
Circles with increasing distance from wound site
Baseline events of GFP-Amph1 punctae in each circle over time
Time (s)
E
Steady-state endocytosis of Dynamin2-GFP in non-wounded cells
Normal time for 'resealing'
Circles with increasing distance from wound site
Baseline events of Dyn2-GFP punctae in each circle over time
Time (s)
F
Before wounding
After wounding
GFP-Amph1
DsRed-CLTA
-1.56 s
1 min 15 s
1 min 53 s
2 min 33 s
GFP-Amph1
DsRed-CLTA
G
AP2σ-GFP
Dyn2-mCherry
-1.56 s
1 min 7 s
1 min 53 s
2 min 33 s
AP2σ-GFP
Dynamin2-pmCherry

Figure S2. Various markers of clathrin-mediated endocytosis show an accumulation to the wound site after PM resealing in HUVEC. (**A**) Scheme showing the quantification strategy used for endocytic punctae count during wound repair. [24] The wounded cell is segmented into 6 concentric circles with increasing distance from the wound site (each circle with increments of 20 μm in diameter from the previous circle). Circle 1 is closest to the laser ablation wound site while circle 6 is the farthest from the wound site. Endocytic punctae are counted in each circle area that is separated from the other circles, and are normalized to the punctae count in the frame before wounding as well as the circle area (due to the varying sizes of the circles). Wound site, red circle on the cell membrane also highlighted by the pink triangle (indicating laser ablation site). (**B and C**) HUVEC were transfected with EGFP-Amphiphysin-1 or Dynamin-2-EGFP and subjected to laser ablation with a low laser power, used as a non-wounding control to study changes in baseline endocytosis. In the absence of membrane damage, the endocytic punctae positive for EGFP-Amphiphysin-1 (**B**) or Dynamin-2-EGFP (**C**) were quantified (as in Figure 1C and D) and represented as heatmaps here. The endocytic punctae were estimated as in the quantification scheme shown in (A) with respect to time after 'wounding' (laser ablation) and represented as counts in each circle normalized to the circle area as well as initial punctae count to study baseline endocytic punctae changes over time. The heatmap colour scaling is matched to the wounded cells' analysis counterparts shown in Figure 1C, D. The green dotted line indicates the normal time required for resealing generally observed in wounded cells. Note that no significant increase in endocytic punctae is seen over time ruling out changes in steady-state endocytosis as a response to membrane wounding (see Figure 1C, D). (**D and E**) Baseline endocytic punctae counts in (B) and (C) are represented as non-normalized quantifications of EGFP-Amphiphysin-1 (**D**) or Dynamin-2-EGFP (**E**) over time in no-wounding controls. Data were not normalized to initial punctae counts and shown for each circle ROI (as per 100 μm^2 of circle ROI area) to further indicate that baseline endocytosis occurs in all parts of the cells and does not show a major change over time. Here, the heatmap colour scaling has been stretched to pick up any bright structures and to ascertain for changes over time. Note that Dynamin2-GFP punctae counts are varied in different circles (circles 2, 3 and 4) possibly owing to its punctate expression seen further in the centre of the cell and contributing to the initial high punctae count (notice the initial time frame and later time frames for each circle). Since increasing circle ROIs have greater circle areas which likely affects the baseline endocytic punctae count comparisons across various parts of the cell, the circle area normalizations have been maintained in the analyses.

Means are plotted for (B), (C), (D) and (E) from 18 – 25 cells pooled from 3 biological replicates. Statistical comparisons were performed to estimate the effect of wounding on endocytic punctae count in different regions of the cell over time by using one-way ANOVA with Kruskal-Wallis test and obtained $P = 0.1229$ for (B), $P = 0.1141$ (C), $P = 0.0646$ (D), and $P = 0.0228$ (E).

(**F**) HUVEC were co-transfected with EGFP-Amphiphysin-1 (green) and the canonical CME coat marker, DsRed-Clathrin light chain A (labelled as DsRed-CLTA; shown in red), and laser injured to study spatial association between the CME markers following membrane damage. Representative time-lapse images before and after injury are shown here. Higher magnifications of the area around the wound site are shown below for each time point (blue boxes). Red triangles mark the laser injury sites and white dashes outline the wounded cells. White arrows indicate the upregulation of GFP-Amph1 punctae after wounding as observed before. The corresponding yellow arrows in the CLTA zoom-in panels show the DsRed-CLTA punctae that concomitantly form close to Amph1 enrichments around the wound site, suggesting spatial association between Amph1 and CLTA events after wounding.

(**G**) HUVEC expressing Dynamin-2-pmCherry (shown in red) and another early-stage marker of CME, AP-2 σ-GFP (green) were laser wounded and subjected to live cell microscopy. Sequential still images are shown here. Zoom-in areas around the wound site are shown below in orange boxes for each protein for the various time points. Red triangles indicate the laser wounding sites. White arrows point to the upregulated Dynamin-2-pmCherry punctae formed around the wound site after resealing. The corresponding yellow arrows label the new AP-2 punctae that have formed around the wound site coinciding with the Dynamin-2 punctae. This suggests that the upregulated endocytic events observed near the wound site following membrane injury are potential endocytic events positive for various CME markers (early-stage markers such as Clathrin light chain A and AP-2, and late stage markers Amphiphysin-1 and Dynamin-2). Note that the high baseline punctate localizations of both early-stage markers prior to wounding do not permit quantification analyses as used for Amphiphysin-1 and Dynamin-2. Images representative of 3 - 4 independent experiments. Scale bars, 10 μm and for zooms, 5 μm.

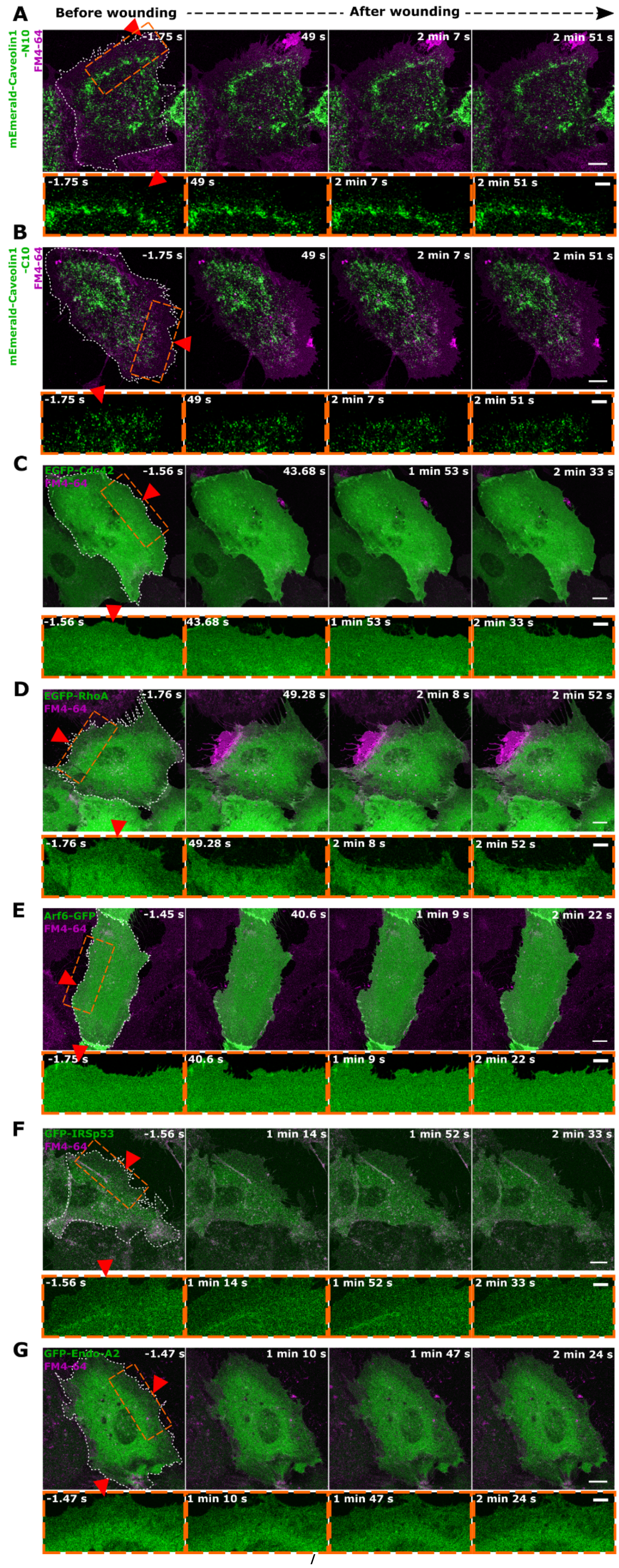
A
Before wounding
After wounding
mEmerald-Caveolin1-N10
FM4-64
-1.75 s
49 s
2 min 7 s
2 min 51 s
B
mEmerald-Caveolin1-C10
FM4-64
C
EGFP-Cdc42
FM4-64
-1.56 s
43.68 s
1 min 53 s
2 min 33 s
D
EGFP-RhoA
FM4-64
-1.76 s
49.28 s
2 min 8 s
2 min 52 s
E
Arf6-GFP
FM4-64
-1.45 s
40.6 s
1 min 9 s
2 min 22 s
-1.75 s
F
GFP-IRSp53
FM4-64
-1.56 s
1 min 14 s
1 min 52 s
2 min 33 s
G
GFP-Endo-A2
FM4-64
-1.47 s
1 min 10 s
1 min 47 s
2 min 24 s

Figure S3. Clathrin-independent endocytosis is not upregulated at the wound site during HUVEC PM repair. (A - E) HUVEC were transfected with the following markers of clathrin-independent endocytic pathways: mEmerald-Caveolin-N-10 (**A**), mEmerald-Caveolin-C-10 (**B**), EGFP-Cdc42 (**C**), EGFP-RhoA (**D**), or Arf6-GFP (**E**) (all displayed in green), and subjected to laser injury in the presence of the membrane-impermeable dye, FM4-64 (magenta). Representative images of the same cell before and after wounding are shown here to assess the dynamics of clathrin-independent endocytosis during membrane wound repair. The wound site is highlighted by the orange box and the corresponding magnifications are shown below for each fusion protein over time. Note the lack of induction of endocytic punctae positive for these marker proteins around the wound site, unlike those observed with markers for clathrin-mediated endocytosis (amphiphysin-1 and dynamin-2) indicating a specific association of CME with HUVEC PM repair. Caveolin-1 localization was tracked using fluorescent reporter constructs tagged at the N- or C-terminus of the protein, in accordance with the reported overexpression artefacts observed in other cells [109] (even though no such phenotype was observed in HUVEC after transfections of these constructs). (**F and G**) HUVEC were transfected with BAR-domain proteins associated with other endocytic pathways, GFP-IRSp53 [47] (**F**) or GFP-Endophilin-A2 (abbreviated as GFP-Endo-A2) [48] (**G**) (both shown in green), and laser wounded in buffer supplemented with FM4-64 (magenta). Still images before and after wounding are shown here. Magnified area around the wound site for BAR-domain protein across various time points is shown below in orange boxes. Note that no GFP-IRSp53 or GFP-Endophilin-A2 structures were observed around the wound site and therefore these proteins do not seem to participate in the upregulated CME observed, as opposed to GFP-Amph1 [29] [30] (see Figure 1A). This also rules out a general accumulation of BAR-domain proteins around the wound site.

Red triangles, wound sites and white dashes, wounded cells. Scale bars, 10 µm and magnifications, 5 µm.

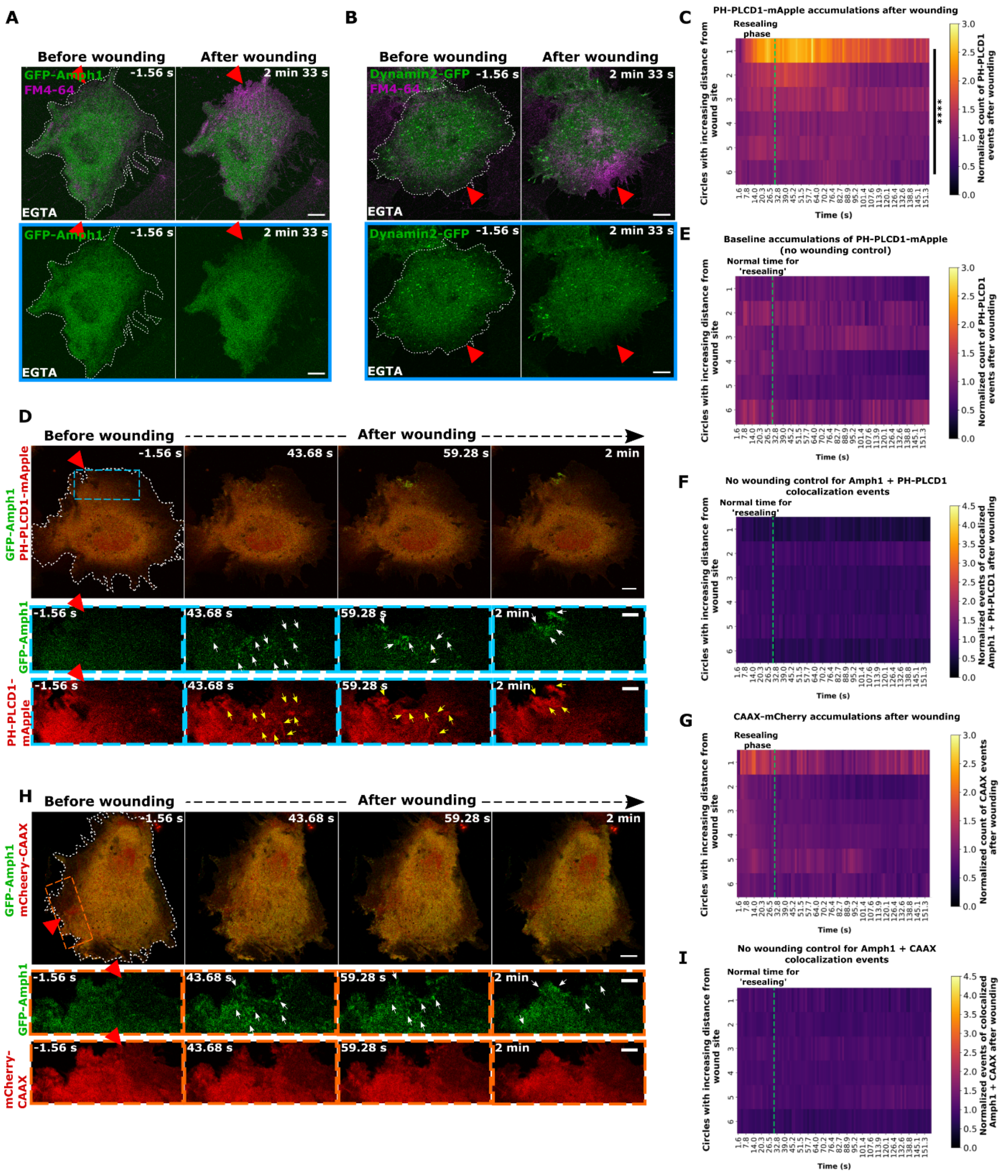

Figure S4. Upregulated CME after wounding is dependent on Ca^{2+}-triggered HUVEC membrane resealing and correlates with PIP_2 accumulations. (**A - B**) HUVEC expressing CME markers, EGFP-Amphiphysin-1 (**A**) or Dynamin-2-EGFP (**B**) were subjected to laser injury in buffer containing the Ca^{2+} chelator, EGTA and FM4-64 dye (magenta and everything else displayed in green). Representative images before and after wounding are shown here. Impaired membrane resealing in the presence of EGTA is revealed by the increase in cellular FM4-64 intensity. No change in the localization of the CME proteins can be observed around the wound site when membrane resealing is impaired in the presence of EGTA. The GFP

channels alone are highlighted below each panel in blue boxes. Images representative of 3 – 4 independent experiments. (**C**) PH-PLCD1-mApple-positive punctae after laser wounding of HUVEC were quantified and represented as heatmaps (as in Figure 1C). The resealing phase in the membrane-wounded cells is indicated by the green dotted line on the heatmap. The punctae counts are normalized to the initial counts before wounding as well as circle ROI area. Note that wounding induces an increase in PIP_2-positive structures immediately after wounding (as observed before [22]) as well as after resealing, correlating with the kinetics of the upregulated Amph1 and Dynamin-2 events. (**D**) HUVEC were co-transfected with EGFP-Amphiphysin-1 (green) and the PIP_2 lipid marker, PH-PLCD1-mApple (shown in red) and laser ablation was performed to assess for any spatial association during wound repair. Representative time-lapse images before and after injury are shown here. Zoomed-in areas around the wound site are shown below for each marker separately for the various time points (blue boxes; see also Figure 1E). White arrows indicate the sites of upregulated GFP-Amph1 punctae after wounding. Yellow arrows point to the corresponding accumulations of PIP_2 occurring at the same sites following wounding indicating colocalized events. (**E**) HUVEC co-transfected with EGFP-Amphiphysin-1 and PH-PLCD1-mApple, were subjected to laser wounding at a low laser power, serving as non-wounding controls and baseline colocalized punctae of both proteins were counted over time. Data were quantified and represented as heatmaps as in Figure 1F. The green dotted line marks the normal time required for resealing in wounded cells (for comparison with punctae counts observed in wounded cells). Note that no changes in stochastically colocalized punctae counts were observed over time in the absence of membrane damage. (**F**) Baseline structures of PH-PLCD1-mApple in non-wounded cells were quantified as in (C) and shown here. Normal time for resealing is marked by the green dotted line on the map. No major changes in random PIP_2 accumulations or structures formed due to lateral diffusion were observed here in contrast to wounded cells (see (C)). (**G**) Normalized punctae counts of mCherry-CAAX in laser wounded HUVEC were quantified and represented as heatmaps. The resealing phase following wounding is indicated by the green dotted line on the map. Note that a few CAAX punctate structures were observed close to the wound site immediately after wounding, potentially owing to the ruffling of the membrane after damage. It is worth mentioning that these structures are much weaker than the PIP_2 accumulations (see (C)) and are not upregulated after membrane resealing (whereas upregulated Amph1 or Dynamin-2 punctae are seen after resealing). (**H**) HUVEC co-expressing EGFP-Amphiphysin-1 (shown in green) and the general membrane marker, mCherry-CAAX (shown in red) were laser injured to asses for spatial association and sequential images are shown here. Higher magnifications of the

wound site are shown below for each marker separately for the different time points (orange boxes; also see Figure 1G). White arrows mark the upregulated GFP-Amph1 punctae after wounding. Note the absence of any corresponding CAAX accumulations following wounding, indicating no association with the enhanced Amph1-endocytic punctae. (**I**) EGFP-Amphiphysin-1 and mCherry-CAAX transfected HUVEC were wounded at a low laser power to study colocalization at these baseline conditions in the absence of wounding. Quantifications were performed and data represented as in Figure 1H revealing no significant colocalization of the two proteins over time under these conditions.

Red triangles, wound site and white dashes, wounded cells. Scale bars, 10 µm, and for magnifications, 5 µm. Means plotted here with $n = 29$ cells for (C), 22 cells each for (D) and (E), 23 cells for (F), and 26 cells for (G). Statistical tests used to analyse punctae count differences across circles were one-way ANOVA with Kruskal-Wallis test with $P < 0.0001$ for (C), $P > 0.9999$ for (D), $P = 0.5267$ (G), and ordinary one-way ANOVA with Tukey's test with $P = 0.8004$ for (E), $P = 0.6709$ (F). $^{****}P < 0.0001$.

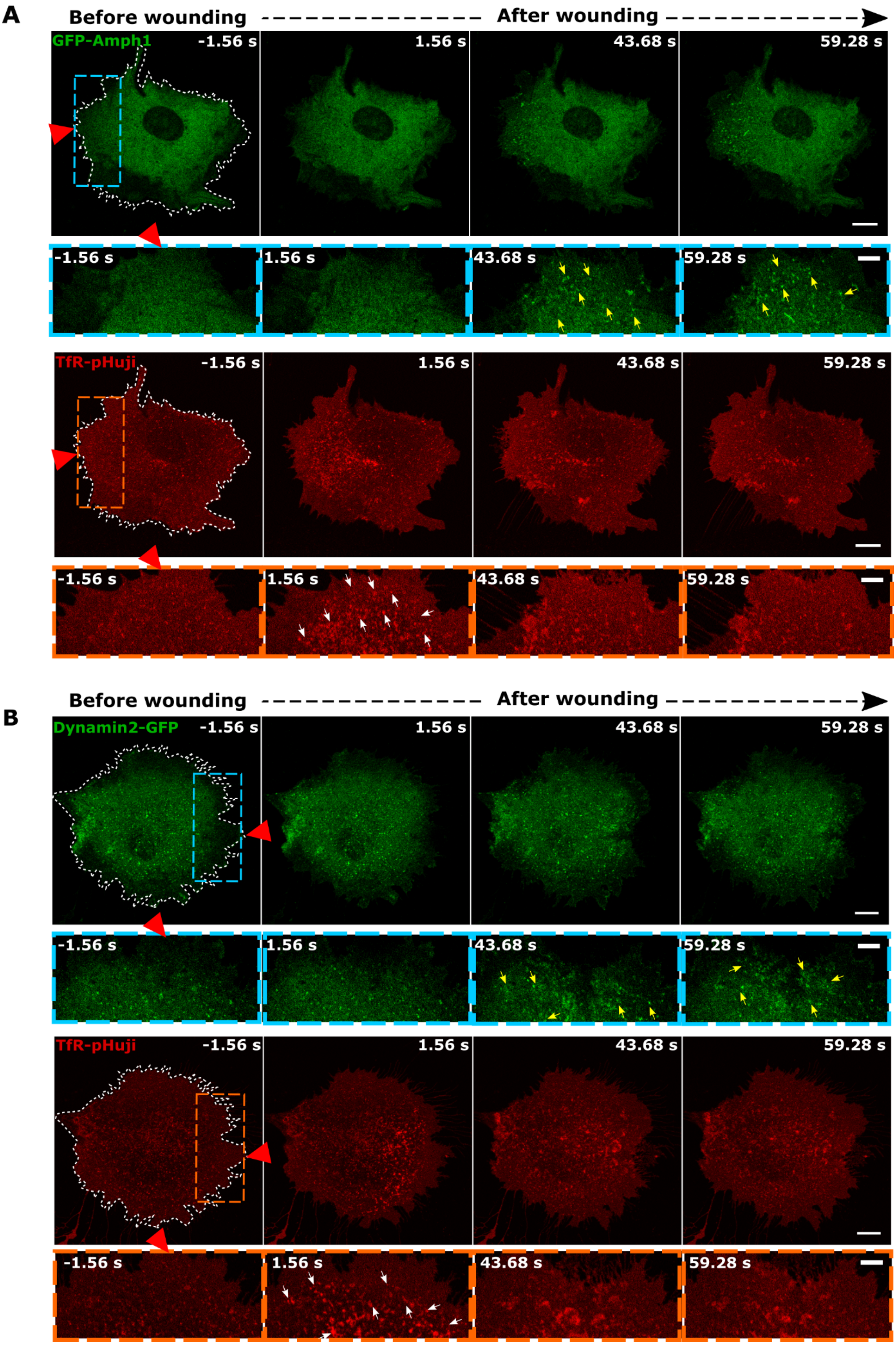
A
Before wounding
After wounding
GFP-Amph1
-1.56 s
1.56 s
43.68 s
59.28 s
TfR-pHuji
B
Dynamin2-GFP

Figure S5. Wound-induced EE exocytosis required for initial membrane repair is spatially associated with wound-induced clathrin-mediated endocytosis occurring at later stages of repair. (**A and B**) Representative single-channel images showing association of early endosome (EE) exocytosis events labelled by an increase of the TfR-pHuji signal (shown in red, bottom panels), with markers of clathrin-mediated endocytosis (CME; shown in green, top panels) after plasma membrane wounding. EGFP-Amphiphysin-1 is used to mark CME in (**A**) (also see Figure 2A) and Dynamin-2-EGFP is employed in (**B**) (also see Figure 2B). Zoom-ins of the wound site are shown under each image as dashed boxes. Blue dashed boxes indicate zoom-in of each time point of CME-associated proteins during wounding and TfR-pHuji images at each time point are magnified in the orange boxes. White arrows indicate sites of TfR-positive exocytosis events occurring immediately after wounding. Yellow arrows mark the CME events occurring at later stages of wounding. Note that the yellow arrows marking CME in (A) or (B) correspond spatially to the white arrows showing TfR exocytosis events which had occurred earlier. This indicates that wound-induced EE exocytosis and CME are spatially associated events. Red triangles, wound sites; wounded cell is outlined in white dashes. Scale bars, 10 μm and for zooms, 5 μm.

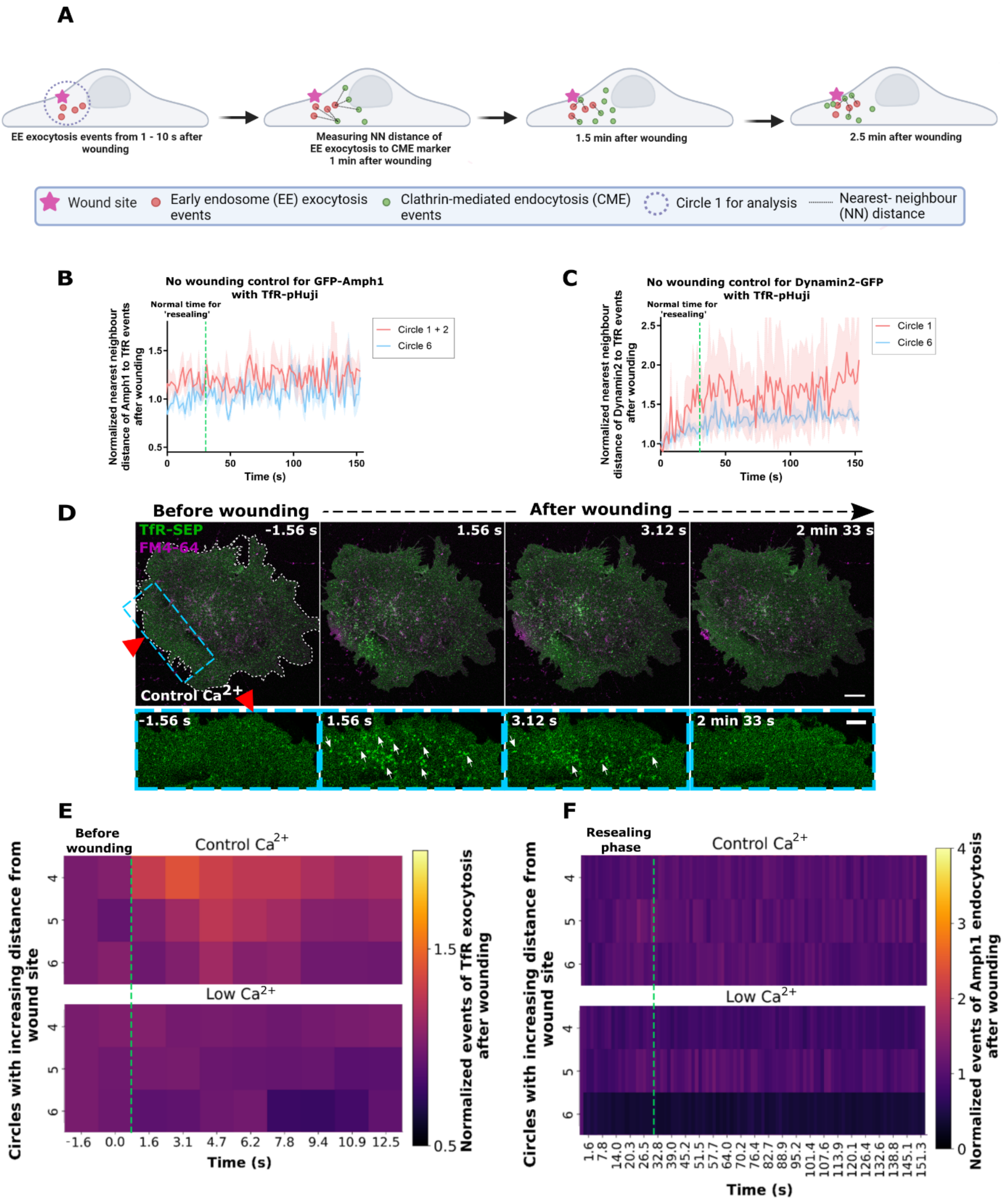

Figure S6. Nearest neighbour analysis of exocytotic and endocytic events to assess their spatial association upon membrane wounding. (A) Schematic showing the nearest-neighbour analysis used to measure the spatial association of EE exocytosis and CME during wound repair. HUVEC transfected with EE exocytosis marker, TfR-pHuji and CME marker (EGFP-Amphiphysin-1 or Dynamin-2-EGFP) were laser injured and time-lapse videos were recorded (as in Figure 2A and B). An image of the TfR-pHuji clusters (appearance of bright fluorescence due to pH neutralization after exocytotic fusion) near the wound site (circle 1) in

the first 10 *s* after wounding was generated to map all EE exocytosis events close to the wound site (marked as red punctae in the animation). The distance of these TfR clusters to all endocytic punctae (shown as green punctae) formed over the entire course of repair (up to 2 min 33 s after wounding) was measured, averaged across various clusters, and calculated as the 'nearest-neighbour' distance (represented in grey lines). If the endocytic punctae formed near the EE exocytosis events, the nearest neighbour distance would be lower and vice versa. The nearest-neighbour distance across all time frames was normalized to the distance before wounding (also see Video S7, Supporting Information; and Experimental Section for further details). Note that depending on the localization and kinetics of the endocytic protein analysed, the initial distance may vary. This is observed in the general increase in NN distance of Dynamin2-GFP to TfR-pHuji punctae in the initial frames after wounding indicating stochastic punctae dynamics (see Figure 2D). (**B and C**) Nearest-neighbour analysis of low laser wounding controls for TfR-pHuji with EGFP-Amphiphysin-1 (**B**) or with Dynamin-2-EGFP (**C**). The green dotted lines on the graphs indicate the normal time required for resealing generally observed in wounded cells, for comparison to the wounded cell counterparts (see Figure 2C, D). Note that the curves for circles near and far away from wound sites do not vary for both endocytic proteins over time. This excludes a role of stochastic association between EE exocytosis and CME events in contributing to the association seen during wound repair. Mean $\pm$ SEM with error bars as the fill area around each curve. n =18 - 20 cells over 3 independent replicates. (**D**) HUVEC transfected with TfR-SEP (green) were laser wounded in buffer containing the wound dye, FM4-64 (magenta) and control levels of Ca^{2+} (2.5 mM). Representative images before and after wounding are shown here, with successful membrane resealing indicated by the FM4-64 dye localization restricted to the wound site. Wound site is magnified below in blue dashed boxes for each time point. White arrows point to TfR-positive early endosome exocytosis events occurring immediately after wounding. [24] Note the drastic change in exocytosis events in the presence of Ca^{2+} as opposed to the low Ca^{2+} results shown in Figure 2H. Red triangles indicate the ablation sites and the wounded cell is outlined in white dashes. Scale bar, 10 μm and for zoom, 5 μm. (**E and F**) Quantifications of punctae count following wounding of TfR-SEP (**E**) and GFP-Amph1 (**F**) expressing cells in the presence of low Ca^{2+} (bottom panel) or control (2.5 mM Ca^{2+}, top panel) are plotted as heatmaps. Only the regions far away from wound site (circles 4 – 6, also see Figure S2A, Supporting Information) are shown here for both conditions (corresponding to the other circles shown in Figure 2I and K). The green dotted line in the heatmap indicates the time point of wounding (E) shown to identify EE exocytosis occurring immediately after wounding and resealing phase occurring around 30 *s* after wounding in (F)

to delineate the endocytic punctae formed in the later stages of repair. Means from 24 – 31 cells over 3 independent replicates. Statistical analysis using two-tailed Mann-Whitney U test was performed for (B) and (C) with $P = 0.4233$ and $P = 0.0596$ respectively, and two-way ANOVA with Tukey's test was done with $P = 0.0610$ for (E) and $P = 0.0234$ (F).

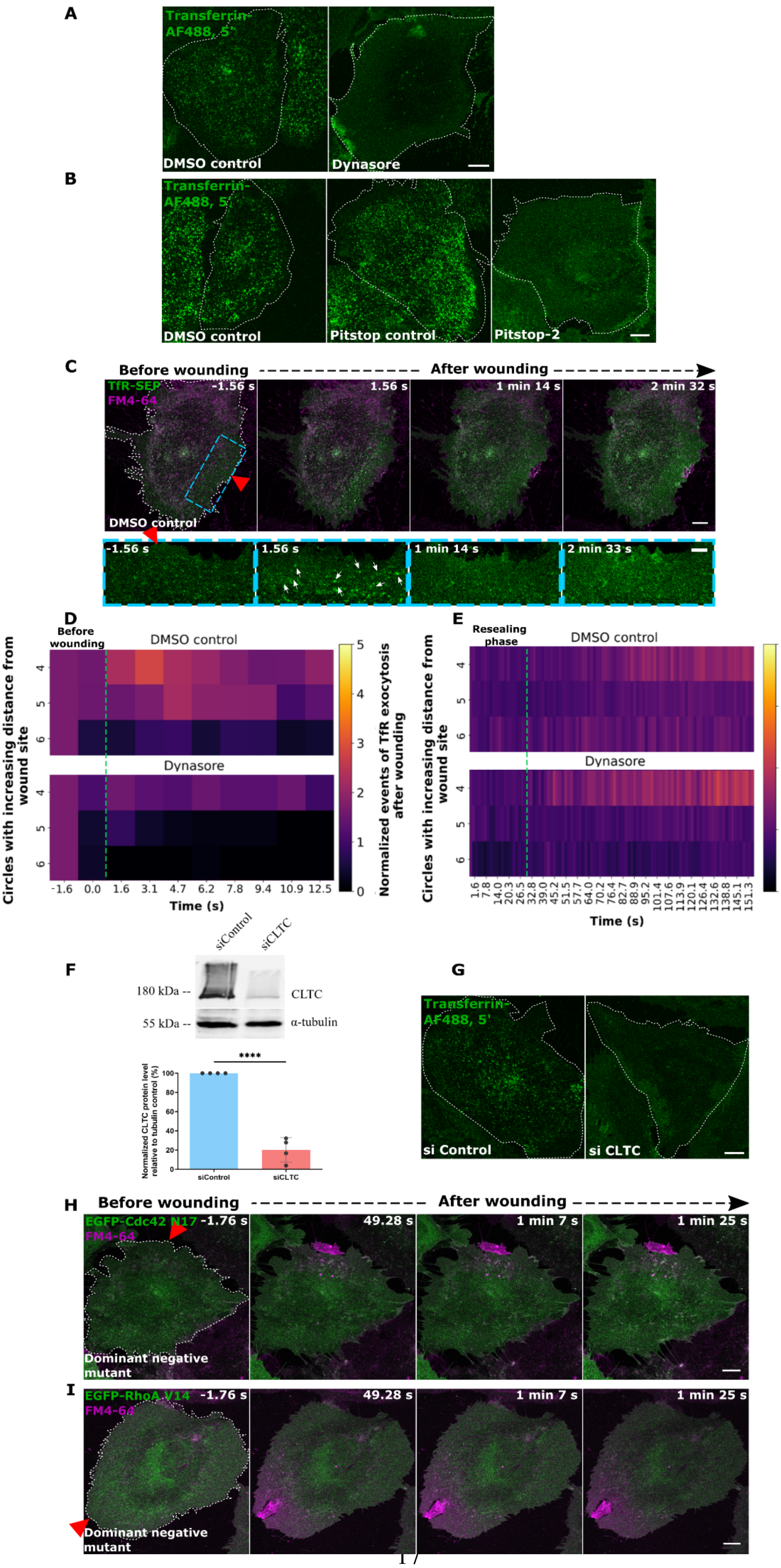
A
Transferrin-AF488, 5'
DMSO control
Dynasore
B
Transferrin-AF488, 5'
DMSO control
Pitstop control
Pitstop-2
C
Before wounding
After wounding
TfR-SEP
FM4-64
-1.56 s
1.56 s
1 min 14 s
2 min 32 s
DMSO control
-1.56 s
1.56 s
1 min 14 s
2 min 33 s
D
Before wounding
DMSO control
Dynasore
Circles with increasing distance from wound site
Normalized events of TfR exocytosis after wounding
Time (s)
E
Resealing phase
DMSO control
Dynasore
Circles with increasing distance from wound site
Normalized events of Amph1 endocytosis after wounding
Time (s)
F
siControl
siCLTC
180 kDa --
55 kDa --
CLTC
α-tubulin
****
Normalized CLTC protein level relative to tubulin control (%)
siControl
siCLTC
G
Transferrin-AF488, 5'
si Control
si CLTC
H
Before wounding
After wounding
EGFP-Cdc42 N17
FM4-64
-1.76 s
49.28 s
1 min 7 s
1 min 25 s
Dominant negative mutant
I
EGFP-RhoA V14
FM4-64
-1.76 s
49.28 s
1 min 7 s
1 min 25 s
Dominant negative mutant

Figure S7. Inhibition of clathrin-mediated and clathrin-independent endocytosis in HUVEC shows varied effects on wound repair. (**A and B**) HUVEC were treated with DMSO (left panels) and CME inhibitors, Dynasore (**A**) or Pitstop-2 (**B**) and the efficiency of endocytic uptake was demonstrated by a short transferrin (Tf) pulse (Transferrin-AF488) for 5 min (shown in green) to label the early endosomal pool. As a negative control for Pitstop-2, the cells were also treated with Pitstop control, which has a similar structure to Pitstop-2 but does not block CME. Representative images of Tf uptake into early endosomes are displayed here. Note the lack of internalized transferrin signal indicating impaired dynamin- and clathrin-mediated endocytosis. (**C**) Laser wounding of HUVEC transfected with TfR-SEP (green) was performed in the presence of FM4-64 (magenta) after treatment with the DMSO vehicle control corresponding to the Dynasore experiments (see Figure 3E). Still images before and after wounding are shown here and the restricted FM4-64 dye localization around the wound site represents efficient membrane resealing. White arrows point to TfR-positive early endosome exocytosis events around the wound site occurring immediately after wounding. [24] Blue dashed boxes indicate the zoomed-in area of the wound site which is shown below for each time point. (**D and E**) Regions far away from the wound site (circles 4 – 6) were quantified over time after Dynasore treatment for: exocytosis marked by TfR-SEP (**D**) and endocytic events labelled by EGFP-Amphiphysin-1 (**E**) and plotted as heatmaps (related to the circles 1-3 shown in Figure 3G and H). Exo- and endocytic punctae counts are normalized for each circle ROI and to the initial events before wounding. The green dotted lines in the heatmaps label the wounding time point (D) and the end of resealing phase in (E). (**F**) Immunoblot showing levels of clathrin heavy chain (CLTC) after siRNA transfection with siControl or pooled siCLTC (top panel) and represented as a percentage of the loading control, α-tubulin here (bottom panel). (**G**) HUVEC were transfected with siControl or pooled siCLTC and the inhibition of endocytosis was assayed as in (A) and (B), by a transferrin pulse for 5 min (shown in green). Note that the siCLTC samples showed an inhibition of endocytic uptake and thus lack of Tf-positive early endosomes. (**H and I**) HUVEC were transfected with the dominant negative mutant of Cdc42, GFP-Cdc42 N17 (**H**, green in image) or the dominant negative mutant of RhoA, EGFP-RhoA V14 (**I**, shown in green as well), and laser wounded in the presence of FM4-64 dye (magenta). The dominant negative isoform of Cdc42 is known to specifically inhibit the clathrin-independent CG pathway [42] while the RhoA V14 isoform inhibits RhoA-dependent endocytic pathways, [123] aiding to evaluate the functional role of these endocytic pathways in HUVEC membrane repair. Representative images after wounding show no defects

in HUVEC resealing as revealed by the confined localization of FM4-64 to the wound site, thus excluding roles for CG and RhoA-dependent pathways in HUVEC PM repair.

Red triangle, wound ROI and white dashes outline wounded cells. Scale bars, 10 μm, and for zooms, 5 μm. Means plotted for (D) and (E) and mean ± SD plotted for (F), pooled from 3 - 4 independent experiments. Statistical analysis was performed with two–way ANOVA with Tukey's test with $P = 0.2161$ for (D) and $P = 0.6102$ (E), and with unpaired two-tailed Student's t-test for (F). ****$P < 0.0001$.

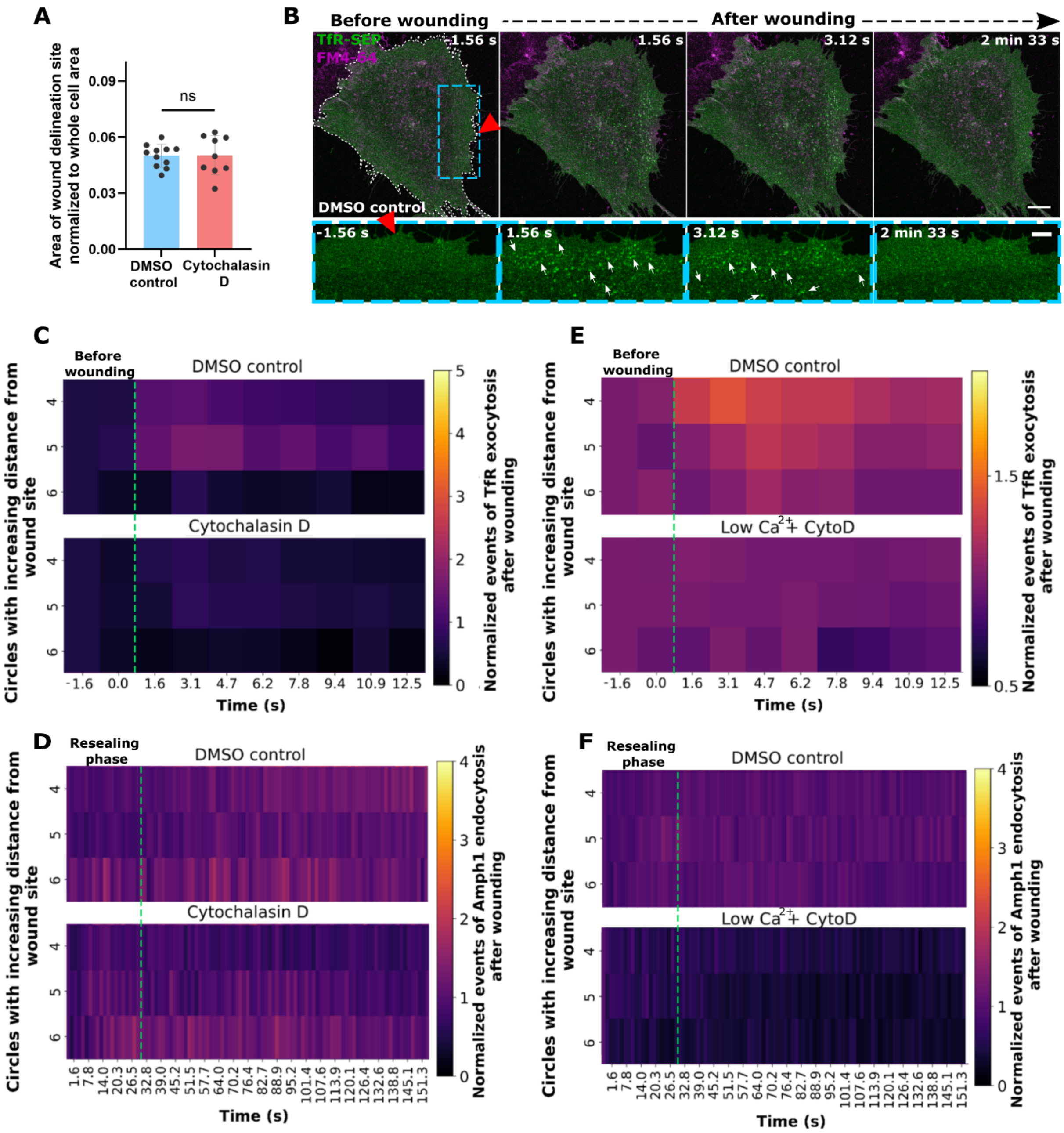

Figure S8. Tension variations do not affect EE exocytosis and clathrin-mediated endocytosis in regions far away from the wound site. (**A**) Membrane resealing efficiency of Cytochalasin D-treated HUVEC after laser wounding was estimated and compared to DMSO control-treated cells. Here, membrane resealing is represented as a measure of the area of FM4-64-marked wound delineation site after membrane repair normalized to the whole cell area. Note that there are no observable differences in wound area in DMSO or Cytochalasin D-treated cells, indicating efficient membrane resealing. (**B**) HUVEC expressing the early endosome exocytosis marker, TfR-SEP (green), were treated with DMSO vehicle control (control for Figure 4D) and subjected to laser wounding assays. Representative time-lapse images during the course of wounding are shown here with wounding indicated by the entry of FM4-64 dye

(magenta). The wound site is magnified for each time point in blue dashed boxes below the whole cell images. White arrows correspond to early endosome exocytosis events marked by an increased TfR-SEP signal around the wound site. [24] Red triangles mark the wound sites and white dashes outline the wounded cells. Scale bar, 10 µm, and for zoom, 5 µm. (**C and D**) Punctae analysis of EE exocytosis events positive for TfR-SEP (**C**) and CME events marked by EGFP-Amphiphysin-1 (**D**) was performed after wounding in DMSO control (top panel of each analysis) or Cytochalasin D-treated HUVEC. The results are plotted as heatmaps over time for circles far away from the wound site (circles 4 - 6) and shown here after normalization to counts prior to wounding and circle area (refer to Figure 4F and G for circles 1-3). The green dotted line indicates the time of wounding for the EE exocytosis count map (C) and the time point of resealing for CME count representation (D). (**E and F**) HUVEC treated with low Ca^{2+} and Cytochalasin D (indicated as CytoD) were wounded and assessed for TfR-SEP count (**E**) or GFP-Amph1 count (**F**), as in (C) and (D). The heatmaps show regions far away from the wound site, revealing no major changes in exo- and endocytosis following treatments (see Figure 4L and M, for circles 1-3).

Mean ± SD and distribution plotted from 17 - 20 cells for (A) and means plotted for all heatmaps from 21 – 31 cells each (C – F). All datasets were pooled from 3 independent experiments. P values were calculated using unpaired two-tailed Student's *t*-test for (A) with P = 0.9467, and two-way ANOVA with Tukey's test showing P = 0.0149 for (C), P = 0.0856 (D), P = 0.0679 (E) and P = 0.056 (F). *ns*, not significant.

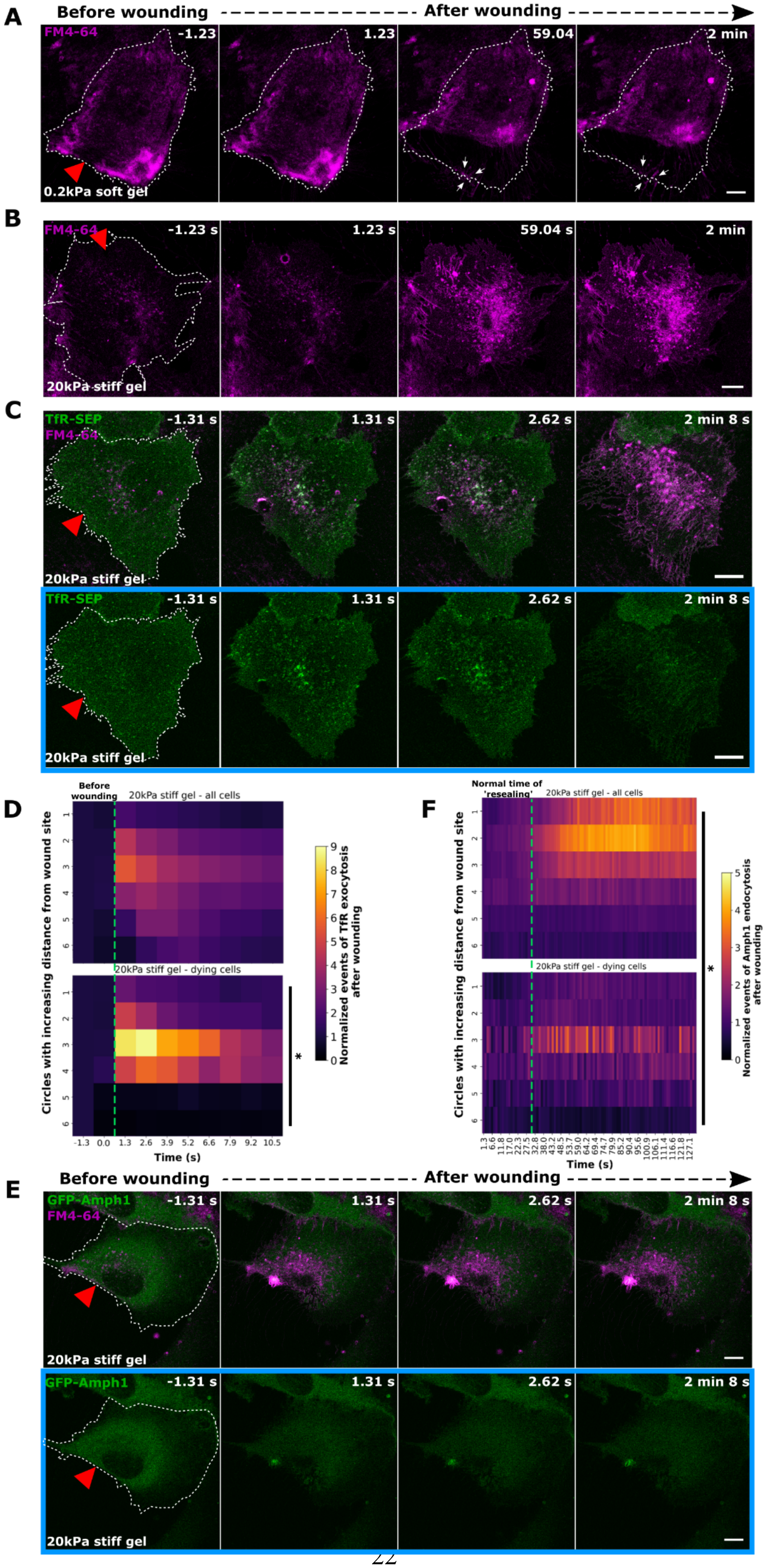
A
Before wounding
After wounding
FM4-64
-1.23
1.23
59.04
2 min
0.2kPa soft gel
B
FM4-64
-1.23 s
1.23 s
59.04 s
2 min
20kPa stiff gel
C
TfR-SEP
FM4-64
-1.31 s
1.31 s
2.62 s
2 min 8 s
20kPa stiff gel
TfR-SEP
-1.31 s
1.31 s
2.62 s
2 min 8 s
20kPa stiff gel
D
Before wounding
20kPa stiff gel - all cells
20kPa stiff gel - dying cells
Circles with increasing distance from wound site
Normalized events of TfR exocytosis after wounding
Time (s)
-1.3 0.0 1.3 2.6 3.9 5.2 6.6 7.9 9.2 10.5
*
F
Normal time of 'resealing'
20kPa stiff gel - all cells
20kPa stiff gel - dying cells
Circles with increasing distance from wound site
Normalized events of Amph1 endocytosis after wounding
Time (s)
*
E
Before wounding
After wounding
GFP-Amph1
FM4-64
-1.31 s
1.31 s
2.62 s
2 min 8 s
20kPa stiff gel
GFP-Amph1
-1.31 s
1.31 s
2.62 s
2 min 8 s
20kPa stiff gel

Figure S9. High membrane tension is associated with compromised membrane repair and reduced EE exocytosis and CME around the wound site in HUVEC. (**A**) HUVEC grown on 0.2 kPa soft gels were laser injured in the presence of extracellular FM4-64 dye (magenta). Still images before and after wounding are shown here. Note the retraction of the entire cell following wounding revealed by the FM4-64 dye labelling of the plasma membrane footprint. This phenotype is observed in 30-40 % of cells grown on soft gels. The white dashes outlining the wounded cell over the time frames after wounding show the footprint of the cell. Also note that the FM4-64 dye influx is still restricted around the wound site (highlighted with white arrows in the image), albeit the lack of attachment of the cell, indicating successful membrane repair in these cells. (**B**) HUVEC cultured on 20 kPa stiff gels were wounded in the presence of FM4-64 dye (magenta) added to the buffer and images before and immediately after wounding are shown. Massive FM4-64 influx into the entire cell indicates defective membrane resealing. Also observe the FM4-64 accumulation at the wound site in the frame after wounding (similar to Figure 5A, bottom panel). Membrane repair was defective in 30-40 % of wounded cells grown on stiff gels. (**C**) HUVEC grown on 20 kPa stiff gels were transfected with TfR-SEP (green) and laser injured in the presence of wounding dye, FM4-64 (magenta). Images after wounding show compromised membrane repair as indicated by the continued influx of FM4-64 dye throughout the cell. Boxed areas below in blue display the GFP channel only (TfR-SEP) for each time point. Note the enhanced EE exocytosis punctae (as indicated by TfR-SEP fluorescence increase) all over the cell. This is probably owed to the open membrane wound in these dying cells which permits sustained Ca^{2+} influx and initiates excess EE exocytosis. (**D**) Quantifications of TfR-SEP exocytosis events after wounding are plotted as heatmaps for all cells grown in 20 kPa stiff gels (top panel) and only for non-repaired cells on 20 kPa stiff gels (bottom panel). Data are normalized and plotted similar to Figure 5G. EE exocytosis close to the wound site is comparable in both populations of stiff-gel cells while the exocytosis events in regions distant from the wound site are upregulated in the dying cells, correlating to a prolonged Ca^{2+} influx through the open wound and a response to deal with the membrane-repair defects observed. (**E and F**) HUVEC were transfected with EGFP-Amphiphysin-1 (green), grown on 20 kPa stiff gels and laser injured in the presence of FM4-64 dye (magenta). Representative still images of a cell showing repair defect after wounding are displayed in (**E**). The Amph1-GFP channel is highlighted below for all the time panels in the blue box. Note the lack of Amph1-endocytic events as opposed to those seen in repairing-competent cells grown on stiff gels, indicating an absence of compensatory endocytosis in non-repaired cells. This is quantified and plotted as heatmaps over time across the entire cell in (**F**) and compared to

Amph1 events in all cells on stiff gels (top panel). No significant Amph1 endocytic events were observable for dying cells grown on stiff gels, suggesting that high tension is detrimental for membrane repair and later restoration of membrane homeostasis.

Red triangle, wound ROI, white outline, wounded cells, and scale bars, 10 µm. Means plotted for all graphs from 3 independent experiments with $n = 12 – 33$ cells (D) and 7 – 27 cells (F) (the cell count for the dying cells corresponds to 30 – 40% of the total stiff gel-grown cells). Statistical comparisons were performed as follows: one-way ANOVA with Kruskal Wallis test to compare punctae count across various regions of the cell in (D, bottom panel) with $P = 0.0203$ and in (F, bottom panel) with $P = 0.4046$, and two-way ANOVA with Tukey's test to compare punctae count in all cells on stiff gels and dying cells on stiff gels with $P = 0.4664$ (D) and $P = 0.0334$ (F). * $P < 0.05$.

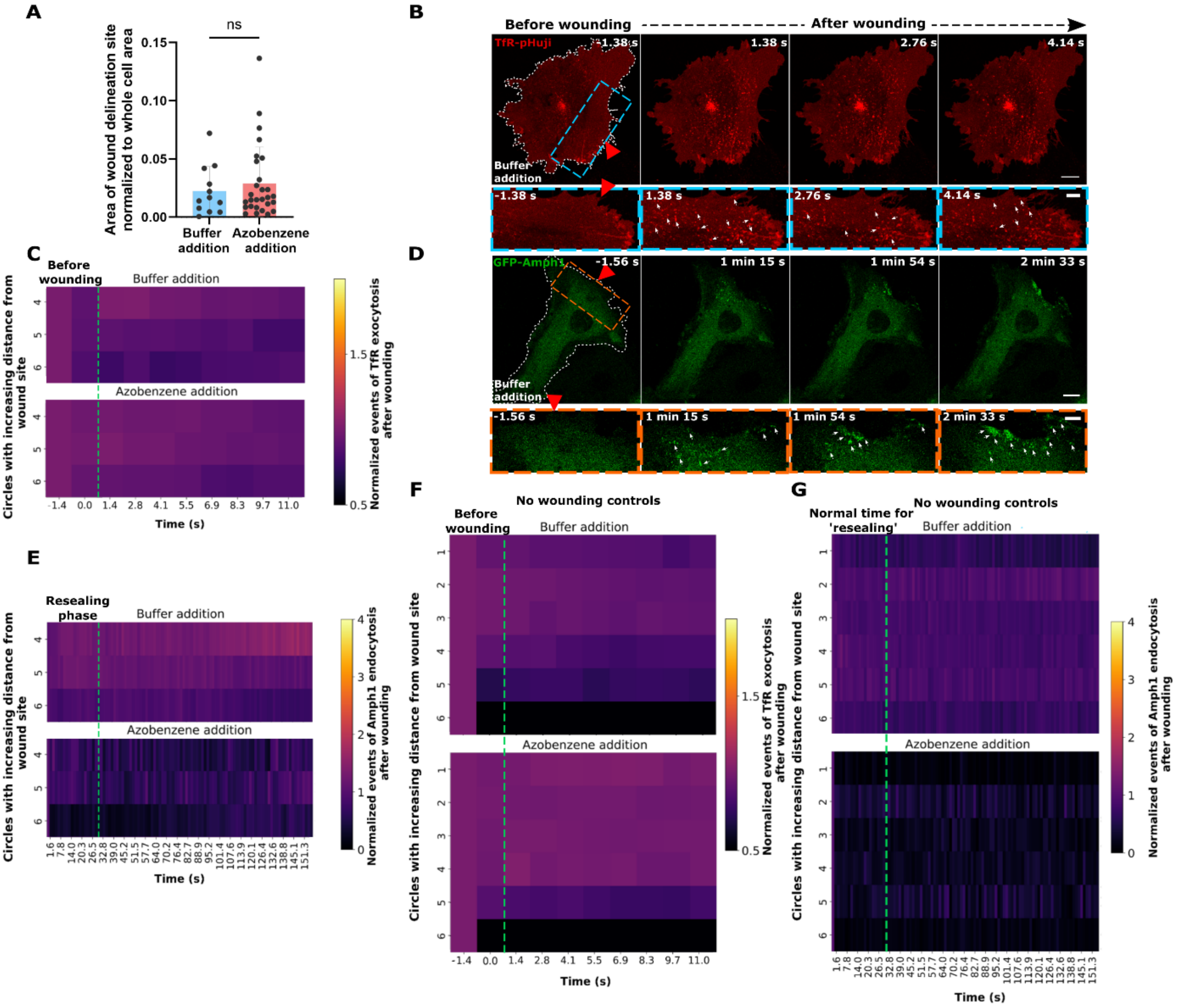

Figure S10. Membrane area increase by azobenzene does not affect EE exocytosis or Amph1-mediated CME in distant parts of wounded cells or in unwounded cells. (**A**) HUVEC kept in buffer control or azobenzene (0.5 mM) were subjected to laser injury and membrane resealing efficiencies were determined. Membrane resealing is quantified here as the area of the wound delineation site (enriched with FM4-64 after ablation) and normalized to the whole cell area. No evident changes in wound area can be seen between control or azobenzene-treated cells, suggesting efficient membrane resealing (also see Figure 6B). (**B and D**) Laser wounding of HUVEC transfected with TfR-pHuji (**B**; shown in red) or EGFP-Amphiphysin-1 (**D**; shown in green) in buffer controls. Still images before and after wounding are shown here with wound areas magnified for each time point below the main panels (blue dashed box for TfR-pHuji and orange dashed box for GFP-Amph1) (also see Figure 6C, D). Red triangles, laser ablation sites, and white dashes outline the wounded cells. White arrows mark TfR-pHuji-positive EE exocytosis immediately after wounding in (B) and Amph1-mediated CME events at later stages of wounding in (D), near the wound sites. (**C and E**) Punctae analysis of EE exocytosis events positive for TfR-pHuji (**C**) was carried out after membrane injury of HUVEC

treated with control (buffer addition, top panel) or azobenzene addition (bottom panel). Punctae analysis of GFP-Amph1 structures occurring after wounding in buffer or azobenzene-treated cells was performed similarly in (**E**). Data are plotted for regions far away from the wound site (circles 4 – 6) over time after normalizations (corresponding graph for circles 1-3 in Figure 6E and F, respectively). The green dotted lines indicate the time point of wounding in (C) and the time point for completion of resealing in (E). (**F and G**) HUVEC kept in buffer alone or azobenzene containing buffer were wounded at a low laser power (non-wounding controls) and TfR-pHuji-positive EE exocytosis counts (**F**) or GFP-Amph1-positive clathrin-mediated endocytic events (**G**) were quantified as in (C) or (E), respectively. Data are plotted for the entire cell (circles 1 – 6) and shown here as heatmaps. No measurable EE exocytotic events or clathrin-mediated endocytic events were noted in contrast to the areas around the wound sites in wounded cells (Figure 6E, F). This excludes an effect of mechanical stimulation that may occur during exchange of solutions (buffer or azobenzene containing buffer) or off-target effects of azobenzene in affecting basal exo- and endocytic processes in cells (without wounding). The green dotted lines in both heatmaps indicate the time of wounding (F) or normal time required for resealing in wounded cells (G).

Mean $\pm$ SD and distribution plotted from 24 - 55 cells for (A) and means plotted from 18 – 21 cells for (C) and (F), 19 – 26 cells for (E), and 16 – 19 cells for (G). All data were pooled from 3 independent experiments. Statistical comparisons were performed using Mann-Whitney U test with $P = 0.5899$ for (A), and two-way ANOVA with Tukey's test for other graphs with $P = 0.2264$ (C), $P = 0.0041$ (E), $P = 0.4857$ (F), and two-way repeated measures ANOVA with Bonferroni's test for (G) with $P = 0.1804$ (G). *ns*, not significant.

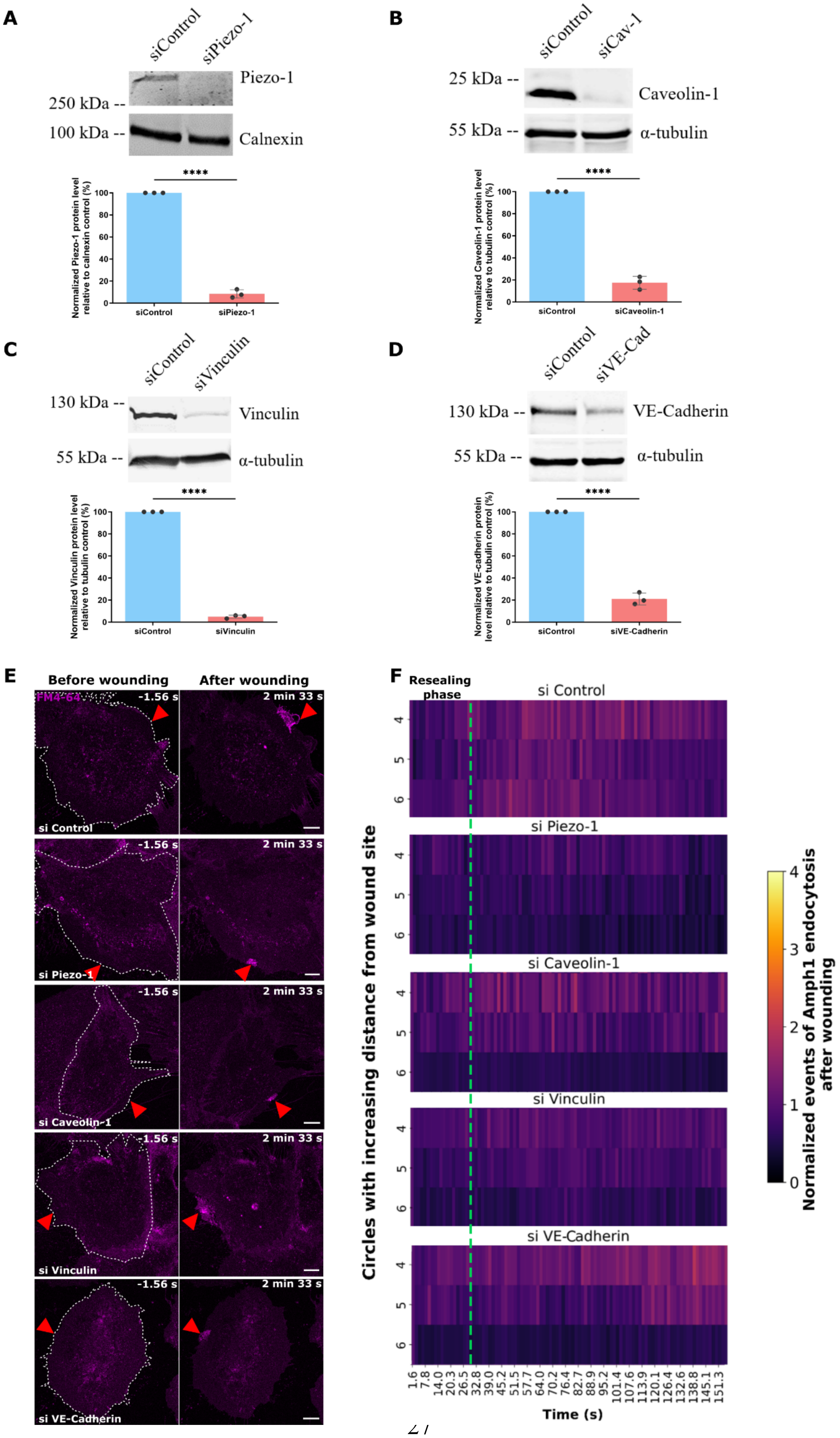
A
siControl
siPiezo-1
Piezo-1
250 kDa --
100 kDa --
Calnexin
****
Normalized Piezo-1 protein level relative to calnexin control (%)
100
80
60
40
20
0
siControl
siPiezo-1
B
siControl
siCav-1
25 kDa --
Caveolin-1
55 kDa --
α-tubulin
****
Normalized Caveolin-1 protein level relative to tubulin control (%)
siControl
siCaveolin-1
C
siControl
siVinculin
130 kDa --
Vinculin
55 kDa --
α-tubulin
****
Normalized Vinculin protein level relative to tubulin control (%)
siControl
siVinculin
D
siControl
siVE-Cad
130 kDa --
VE-Cadherin
55 kDa --
α-tubulin
****
Normalized VE-cadherin protein level relative to tubulin control (%)
siControl
siVE-Cadherin
E
Before wounding
After wounding
FM4-64
-1.56 s
2 min 33 s
si Control
si Piezo-1
si Caveolin-1
si Vinculin
si VE-Cadherin
F
Resealing phase
si Control
si Piezo-1
si Caveolin-1
si Vinculin
si VE-Cadherin
Circles with increasing distance from wound site
4
5
6
Time (s)
1.6
7.8
14.0
20.3
26.5
32.8
39.0
45.2
51.5
57.7
64.0
70.2
76.4
82.7
88.9
95.2
101.4
107.6
113.9
120.1
126.4
132.6
138.8
145.1
151.3
Normalized events of Amph1 endocytosis after wounding
0
1
2
3
4

Figure S11. Depletion of different mechanosensor proteins in HUVEC does not affect membrane resealing but inhibits endocytosis at later stages of membrane repair. (**A - D**) HUVEC were transfected with siControl or siRNAs against different mechanosensitive proteins and knockdown efficiencies were assessed by Western blots of total lysates probed with the respective antibodies directed against Piezo-1 (**A**), Caveolin-1 (**B**), Vinculin (**C**) and VE-Cadherin (**D**). Calnexin (A) or α – tubulin (B – D) were used as loading controls (top panels). The graph at the bottom of each panel shows the analysis of the knockdown efficiency of each siRNA, plotted as a percentage normalized to the loading control. Significant depletion of all mechanosensitive proteins was observed. (**E**) HUVEC membrane resealing was analysed after depletion of the different proteins shown in (A – D). Following siRNA transfection, cells were laser injured in the presence of FM4-64 (magenta). No major defect in membrane resealing was observed in all cases, indicated by the delineation of the FM4-64 dye influx to the ablation site similar to the control sample. Red triangles, ablation sites. White dashes, cell outlines. Scale bars, 10 µm. (**F**) HUVEC transfected with siRNAs targeting the different mechanosensor proteins (as shown in (E)) and co-transfected with EGFP-Amphiphysin-1 were analysed for CME events after wounding. Punctae counts in regions far away from the wound site (circles 4 – 6) are plotted as heatmaps (circles 1-3 shown in Figure 6L). The green dotted line indicates the completion of the resealing phase. No marked change in Amph1 endocytic punctae was observed for all samples in varying parts of the cells.

Mean ± SD plotted for 3 independent experiments for (A – D) and means plotted for (F) from 21 – 27 cells combined from 3 biological replicates. Unpaired two-tailed Student's *t*-test was performed for (A – D) with $P < 0.0001$. For the comparisons of punctae count of each siRNA transfection with siControl in (F), two-way ANOVA with Tukey's test was utilized with P = 0.0823 (Piezo-1), 0.2765 (Caveolin-1), 0.2028 (Vinculin), and 0.5822 (VE-Cadherin). **** P < 0.0001.

Video legends

Video S1.

Time lapse recording of laser ablation of HUVEC shows the appearance of CME punctae (EGFP-Amphiphysin-1, green) after membrane resealing (FM4-64 wounding dye in magenta), related to Figure 1A. Red triangle, wound ROI. White arrows indicate Amph1 punctae that appear after wounding in the later stages of repair. Frames were captured at 1.56 *s* intervals. Time is displayed in minutes and t = 0 *s* represents the time of wounding. Scale bar, 10 μm.

Video S2.

Time lapse recording of laser ablation of HUVEC shows the appearance of CME punctae (Dynamin-2-EGFP, green) after membrane resealing (FM4-64 wounding dye in magenta), related to Figure 1B. Red triangle, wound ROI. White arrows indicate Dynamin-2 punctae that appear after wounding in the later stages of repair. Frames were captured at 1.56 *s* intervals. Time is displayed in minutes and t = 0 *s* represents the time of wounding. Scale bar, 10 μm.

Video S3.

Time lapse recording of laser ablation of HUVEC shows the appearance of Amph1 punctae (EGFP-Amphiphysin-1, shown in green) close to the wound site after wound resealing that colocalize with the appearance of clathrin light chain punctae (DsRed-Clathrin light chain A, displayed in red), during the later stages of repair (related to Figure S2F, Supporting Information). Red triangle, wound ROI. White arrows indicate the spatial association of upregulated Amph1 CME punctae with the new CLTA punctae around the wound site. Fresh CLTA events that appear around the wound site are highlighted again with yellow arrows due to the punctate background. Images were taken every 1.56 *s*. Scale bar, 10 μm.

Video S4.

Time lapse recording of laser ablation of HUVEC shows the appearance of Dynamin-2 punctae (Dynamin-2-pmCherry, displayed in red) that colocalize with the appearance of AP-2 punctae (AP-2 σ-EGFP, green) close to the wound site during the later stages of repair (related to Figure S2G, Supporting Information). Red triangle, wound ROI. White arrows indicate the spatial colocalization of Dynamin-2 punctae with the new AP-2 events formed around the wound site. Images were taken every 1.56 *s*. Scale bar, 10 μm.

Video S5.

Time lapse recording of laser ablation of HUVEC shows the appearance of clusters of cell surface transferrin receptor (TfR-pHuji, red, increased fluorescence after exocytosis-induced pH neutralization) immediately after membrane wounding and the subsequent appearance of CME (EGFP-Amphiphysin-1, green) in the later stages of repair (related to Figure 2A). Red triangle, wound ROI. White arrows indicate the spatial association of Amph1 CME events with the TfR clusters post wounding. Images were taken every 1.49 *s*. Scale bar, 10 µm.

Video S6.

Time lapse recording of laser ablation of HUVEC shows the appearance of transferrin receptor (TfR-pHuji, red) clusters immediately after membrane wounding and the subsequent appearance of CME (Dynamin-2-EGFP, green) in the later stages of repair (related to Figure 2B). Peripheral TfR-pHuji exocytosis events (occurring at the edges of the cell which are close to the plasma membrane) which occur upon wounding have been observed before [24]. This is more evident in this cell as it is less flat as compared to other HUVEC and therefore the TfR-pHuji events seems to cover a larger region of the cell (reflecting the biological variability in cells). Red triangle, wound ROI. White arrows indicate the spatial association of Dynamin-2 CME events with the TfR clusters post wounding. Images were taken every 1.49 *s*. Scale bar, 10 µm.

Video S7.

Video showing an example of the nearest neighbour analysis used to measure the spatial association of EE exocytosis events near the wound site (in circle 1) (TfR-pHuji segmented punctae, red) and clathrin-mediated endocytosis (EGFP-Amphiphysin-1 segmented punctae, green) after membrane wounding. Red triangle, wound ROI. Circle 1 is marked by the white concentric circle covering the cell. Blue lines indicate the distances measured between the EE exocytotic punctae in circle 1 and all the Amph1 punctae. White dashed lines outline the entire cell in the first 15 frames of the video. Note that the TfR-pHuji punctae shown reflect absolute punctae following segmentation without normalizing for baseline bright structures which account for the punctate distribution in a larger region of the cell (observe the dashed cell outline). The mapping is performed prior to the normalizations and thus shown here for transparency. Video taken with a frame interval of 1.56 *s* is shown for the TfR punctae combined from the first 10 *s* of wounding and the nearest-neighbour analysis for Amph1 is shown for the entire time series. Scale bar, 10 µm.

Video S8.

Time lapse recording of laser ablation of HUVEC grown on 0.2 kPa soft gels shows efficient membrane resealing (FM4-64 dye in magenta) (related to Figure 5B, top panel). Red triangle, wound ROI. White arrows indicate the delineation of the wound resealing site in the later stages of repair. Frames were captured at 1.31 *s* intervals. Time is displayed in minutes and t = 0 *s* represents the time of wounding. Scale bar, 10 µm.

Video S9A.

Time lapse recording of laser ablation of HUVEC grown on 20 kPa stiff gels shows delayed membrane resealing (FM4-64 dye in magenta) (related to Figure 5B, bottom panel). Red triangle, wound ROI. White arrows indicate the large wound resealing site indicating the enhanced FM dye influx and delayed repair. Frames were captured at 1.31 *s* intervals. Time is displayed in minutes and t = 0 *s* represents the time of wounding. Scale bar, 10 µm.

Video S9B.

Time lapse recording of laser ablation of HUVEC grown on 20 kPa stiff gels shows inhibited membrane resealing (FM4-64 dye in magenta) (related to Supplementary Figure S9B). Red triangle, wound ROI. Frames were captured at 1.31 *s* intervals. Time is displayed in minutes and t = 0 *s* represents the time of wounding. Scale bar, 10 µm.
